# Exploring depression symptoms in chronic care users in Dr Kenneth Kaunda for culturally relevant counselling

**DOI:** 10.4102/phcfm.v17i1.4958

**Published:** 2025-09-23

**Authors:** One M. Selohilwe, Tasneem Kathree, Arvin Bhana, Inge Petersen

**Affiliations:** 1 Centre for Research in Health Systems, School of Nursing and Public Health, University of KwaZulu-Natal, Durban, South Africa

**Keywords:** contextually relevant interventions, depression, lay-health workers, South Africa, task sharing

## Abstract

**Background:**

South Africa is faced with a mental health burden attributed to a large treatment gap for common mental disorders (CMDs), and a shortage of mental health professionals. Although comorbidity of CMDs with chronic diseases is common, chronic and non-communicable diseases may receive more attention than CMDs highlighting the need for contextually appropriate, culturally relevant counselling to increase access to mental healthcare for CMDs at primary health care (PHC).

**Aim:**

To explore the experiences of patients with comorbid chronic medical conditions and depression attending PHC, to inform the adaptation of an existing evidence-based lay counselling intervention developed in South Africa for human immunodeficiency virus (HIV)-positive patients.

**Setting:**

Dr Kenneth Kaunda district, North West province, South Africa.

**Methods:**

Semi-structured qualitative interviews were conducted with 16 Sestwana speaking adult chronic care patients with hypertension and HIV who screened positive for depressive symptoms using the Patient Health Questionnaire (PHQ-9), to explore their lived experiences of depression.

**Results:**

Poor understanding of depression and poor mental health literacy were highlighted. Depressive symptoms were commonly associated with social determinants including poverty, interpersonal conflict, stigma, illness and grief and bereavement. Most participants were unaware of available depression treatments.

**Conclusion:**

Psychoeducation to improve mental health literacy, cognitive behavioural interventions and problem-solving techniques using task sharing are recommended.

**Contribution:**

There is limited evidence of explanatory models for depression among this population in South Africa. To our knowledge, this is the only study that focused on a predominantly Setswana-speaking chronic care adult population with comorbid depression.

## Introduction

Depression is a leading cause of disability affecting an estimated 332.41 million people worldwide and accounts for the highest percentage of disability adjusted life years (DALYS) (36.24%).^1^ Depressive disorders are particularly high in sub-Saharan Africa (4540.4 cases per 100 000 people).^2^ Estimates from research show approximately 27% of South Africans have probable depression,^3^ with an estimated 30.3% of the adult population experiencing a common mental disorder (CMD) (depression, anxiety or a substance use disorder) over the course of a lifetime.^4^ Depression is the leading individual disorder, having a lifetime prevalence of 9.7%.^5^

A large proportion of people in South Africa with mental health conditions do not receive the treatment they need, with an estimated treatment gap of 92%.^6^ A paucity of human resources and expertise for mental health care contributes to this service delivery gap.^6,7,8^

Poor mental health literacy contributes to poor demand for services.^9^ In addition to service quality and accessibility, patients’ knowledge can have an impact on how much they gain from available mental health treatments.^10,11^ Mental health literacy can be understood as knowledge and beliefs an individual holds regarding mental problems that help with their detection and management or prevention.^12^ This includes: knowing how to look for mental health information, recognising specific conditions, being aware of risk factors and causes, using self-care techniques, being aware of professional help that is available and having attitudes that support these behaviours.^12^

People living with chronic medical conditions are vulnerable to having comorbid depression, which is associated with poorer health outcomes, increased health costs because of complications, increased physical disability and increased health care utilisation.^13,14,15^ Services to treat depression in patients with chronic conditions are thus important to contain these negative impacts. The large treatment gap for mental health conditions is a concern.

In response to the rising burden of multimorbid long-term conditions presenting in the South African population, the National Department of Health in South Africa has adopted an Integrated Clinical Services Management (ICSM) model to promote person-centred integrated care, as well as improve continuity of care over time.^16,17,18^ The integration of mental health into primary health care (PHC) is part of ICSM and provides the opportunity to ensure that mental health care is part of integrated services from the outset of restructuring PHC services as opposed to being added on. Such integrated services should include access not only to psychotropic medication where necessary but also to psychological treatments. Both have been shown to be effectively delivered by non-specialists through a task-sharing approach.^19,20^

Task sharing is a process whereby general health workers are trained to provide services in order to increase capacity and health care accessibility in low-resource settings^21^ and is embraced by the South African Mental Health Policy Framework and Strategic Plan (2013–2020).^22^ The effectiveness of task-sharing psychological treatments using cognitive-behavioural interventions and interpersonal therapy (IPT) is now well established internationally.^19^

In South Africa, task sharing has also been mooted as potentially helping with providing context-relevant interventions, as well as bridging the language-culture gap that often exists between service users and professionals.^23^ The need for translational research to adapt evidence-based psychological interventions for particular socio-cultural contexts is, however, essential.

Against this background, the aim of this study was to understand the experience of depressive symptoms of chronic care patients (inclusive of communicable and non-communicable diseases) in a low-resourced South African district. This was with the view to informing the adaptation of an existing group-based counselling intervention previously designed for depression in human immunodeficiency virus (HIV)-positive patients for use with an expanded population of chronic care patients with depressive symptoms^24^ so that the intervention could be available to all patients in the chronic stream given the rising burden of non-communicable disease.^25^

Our specific research questions were:

- *How do people with chronic conditions understand the phenomenon of depression, particularly in terms of its manifestations and course?*
- *What influences do service users living with chronic conditions identify as a source of their depression?*
- *What interventions do they perceive as helpful?*

The adapting interventions to new contexts (ADAPT) guidance helped provide a framework for careful and systematic adaptation and recommends in-depth understanding of the needs of end-users and their context for intervention adaptation.^26^ The first step of the guidance was utilised for purposes of this study. The first step deals with assessing the rationale for the intervention and giving consideration to the intervention context fit.^26^

## Research methods and design

### Conceptual framework

Exploratory descriptive qualitative studies offer a means for understanding complex and nuanced phenomena in various fields, particularly within the healthcare domain. Through this method, rich nuanced insights that represent participants’ lived experiences can be gathered, allowing for deeper engagement with the human elements of health and illness that quantitative studies may overlook.

We employed Kleinman’s conceptualisation of explanatory models of illness to guide the interview questions.^10^ An explanatory model of illness seeks to understand how personal, social and cultural influences shape how a group of people within a particular socio-cultural context understand and label their illness, its cause, course and treatment. There is growing evidence on explanatory models of depression in different settings in Africa to ensure identification and treatment are both context- and culturally appropriate.^27^

### Setting

The study was conducted in 2013 in the Dr Kenneth Kaunda (DKK) in the North West Province. The Dr KK district had 27 PHC facilities, nine community health centres, three district hospitals and one regional hospital servicing approximately 700 000 people at the time of the study.^28^ Two district psychologists offered in-reach services at the PHC clinics, where PHC services were provided primarily by nurses and rotating medical officers. The district hospitals also had a referral outpatient psychology clinic. The study site from where the sample was drawn was a community health centre serviced by nursing staff and rotational medical officers servicing approximately 78 400 people at the time of the study.^29^

### Study population

This research focused on adult service users attending the aforementioned Community Health Centre (CHC) who had a chronic physical condition and who were screened as also having depressive symptoms.

Inclusion criteria included service users 18 years and above, who were attending a routine chronic care clinic, and understood Setswana or English.

### Recruitment

A purposive sampling approach was used. Participants were recruited from the PHC facility’s waiting room assigned for chronic care patients for 1 week. A brief explanation of the study was provided in the waiting room, and participants were invited to take part in the study. Willing participants provided verbal pre-consent to be screened by a bilingual English/Setswana-speaking psychologist (OS) using the Patient Health Questionnaire (PHQ-9), which is a screening tool for depression, previously validated for use in PHC settings in South Africa, with a cut-off of 10^30^ and also since validated for the Setswana-speaking population.^31^ Participants who screened positive on the PHQ-9 were enrolled in the study after informed consent procedures, resulting in 16 female participants. Men were not excluded, but evidence shows that women use more PHC services than men in South Africa.^32^

### Data collection

Qualitative data for this formative work were collected over 1 week through semi-structured in-depth interviews with service users using an interview schedule conducted by a bilingual psychologist (PMM) fluent in Setswana and English. The interview schedule sought to elicit service users’ experience of depression and their understandings of the causes, symptoms, course and treatments using open-ended questions with a focus on understanding participants’ experiences from their perspectives to allow the participants as experts of their own lives to narrate their stories. The interviews were audio-recorded, were approximately 1 h and took place in a private room at the participant’s primary health institution. Transcripts were not returned to the participants.

Participants were interviewed in their preferred language, and all participants in the study chose to be interviewed in Setswana.

### Data analysis

After the transcripts were translated and transcribed, the data were thematically analysed using Braun and Clarke’s^33^ thematic analysis methodology (2006) employing NVivo 11 qualitative software package. The steps included: (1) acquainting oneself with the data by reading and re-reading the data, (2) organising the data in a meaningful and systematic way, (3) examining and collating the data into potential themes, (4) reviewing themes, (5) coding data according to the themes and (6) producing the report.

In order to ensure a thorough examination of themes within Kleinman’s explanatory model of illness, the transcripts were analysed using a hybrid approach that integrated deductive and inductive coding with theme development.^34^ During the process of coding themes, the researchers also kept a record of ideas and insights emerging, as well as possible interpretations that we returned to in subsequent phases. The team reached a consensus on the final themes.

### Reflexivity

P.M.M. who conducted the interviews and O.M.S. who analysed the interviews were both clinical psychologists. They had to reflect on whether their professional background may have influenced the participants’ willingness to discuss their experiences honestly or if it shaped their responses in particular ways. Open-ended questions that focused on understanding participants’ experiences from their perspectives and the use of micro-counselling techniques during the interview allowed participants to be experts on their own lives so as to narrate their stories. O.M.S., in analysing the data, was deliberate about setting aside any preconceived ideas on the experience of depression so as to have an unbiased view of the participants’ responses in relation to their experience of depression.

### Ethical considerations

Ethical approval was obtained from the University of KwaZulu-Natal Biomedical Research Ethics Committee (BREC) (ethical clearance number: HSS/0880/011; BE258/14). Participants provided written consent to be included in the study. Participants who showed distress or suicidal ideation by expressing a death wish or talking about wanting to kill themselves during the interview were immediately referred to the consulting nurse for immediate management. The consulting nurse was also alerted about all other participants who had mild to moderate depression. All participants were also issued with information on available telephonic assistance. All procedures performed in studies involving human participants were in accordance with the ethical standards of the institutional ethics committee and with the 1964 Helsinki Declaration and its later amendments or comparable ethical standards.

## Results

All the participants were female, and over 50% of them did not have a partner. None of the participants had formal employment, and half were dependent on state social welfare (Table 1).

**TABLE 1:** Demographic characteristics of the sample.

Demographic characteristics	*N* = 16
**Gender**	
Female	16
**Marital status**	
Married	5
Single	3
Has a partner	1
Widow	5
Divorced	1
Missing data	1
**Employment status**	
Self-employed	2
Pension/grants	8
Unemployed	7
Housewife	1
Missing data	2

A total of five main themes emerged from the data analysis, and these were: (1) understanding depressive symptoms (symptoms of depression), (2) perceived causes of depressive symptoms and exacerbating factors (poverty and financial difficulties, worry, interpersonal conflict, death, traumatic life experiences and ill health, social isolation and self-stigma and low self-esteem), (3) help seeking, (4) healing and recovery, and (5) possible interventions and attitudes to task sharing.

### Theme 1: Understanding of depressive symptoms

Many participants reported that they did not know what ‘depression’ was. Those who reported knowing about depression defined it as ‘stress or tired nerves’ and as a result of painful emotional experiences or difficult times. Although the concept of depression was not known to some of the participants, they could all talk about their experiences of the symptoms and their impact on their lives.

#### Symptoms of depression

Participants reported the following: low mood, social isolation, hopelessness and talk of death, worry, anhedonia, anger and irritability, memory problems and a lack of concentration, crying, stress, depersonalisation and being violent. Participant 7 talked about memory problems and a ‘heart’ that is constantly aware of the pain her husband has caused her:

‘I have times when I speak to myself. I forget a lot of things. Sometimes I struggle to fall asleep or wake up in the middle of the night and I would try to pray to forget but I see that my heart doesn’t want to forget.’ (Participant 07, F, unemployed)

Physical symptoms associated with depression were reported by some participants, and these included experiencing weight loss, decreased energy and tiredness, headaches and general body pain. Participant 02 talked about how the disorder incapacitated her – her mind and body being in a state of ‘not being able to do anything’, impacting functioning:

‘… I don’t see the use when I’m struggling like this because sometimes my body goes into a state of inability …’ (Participant 02, F, unemployed)

Although most participants could articulate their symptoms of depression, they did not attribute this to being depressed or know that they were suffering from depression. Their reported lived experiences of symptoms support the universality of depressive symptoms. The participants described their understanding of the symptoms using contextually informed metaphors and explanations.

**FIGURE 1:**
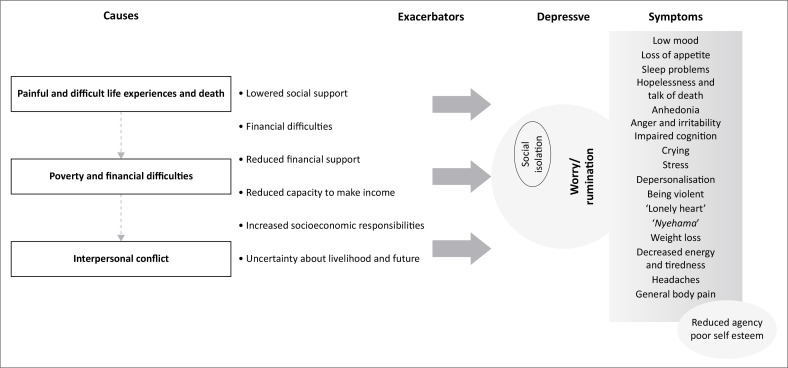
A figure showing causal influences and exacerbators of depression in the study sample.

Participants’ understanding of depression was relayed mainly through the embodiment of symptoms, and in many cases, the heart was construed as the seat of emotions and talked about bodily symptoms, showing how adverse and distressing events cause worry (‘overthinking’, ‘talking with heart’) and impact on the body and everyday functioning (see Figure 1).

### Theme 2: Perceived causes of depressive symptoms and exacerbating factors

Participants identified five main causal factors of depression, namely poverty and financial difficulties; worry; interpersonal conflict; death, painful life experiences and ill health; and social isolation and self-stigma.

#### Poverty and financial difficulties

The experience of poverty and financial strain, difficilties and problems were reported as the main triggers od depression by most participants, who reported challenges including struggling to support themselves and their children. Children left behind by loved ones who had passed away added to the financial strain. Unemployment and a lack of consistent income were also reported as causal factors for depression.

Worrying about the lack of housing or the threat of being dispossessed of a home was reported as an exacerbating factor for depression by some of the participants. Participant 14 discussed how an unemployed status and a continuous lack of money to provide for the families made the situation progressively worse:

‘[*Depression*] is brought on by unemployment, struggling without any income whatsoever. That is the main cause, having children and not having an income.’ (Participant 14, F, unemployed)

#### Worry

‘Overthinking’ or ‘thinking a lot’ and ‘talking with your heart’ or ‘carrying something in your heart’ or ‘talking alone’ were reported as both causing and exacerbating depressive symptoms, especially when related to worries about livelihood. Worry was reported as a causal factor for depression, and many people said worry made their days worse:

‘[*Depression is caused by*] taking time to think about your financial needs; you don’t have a job or anyone to turn to. When you have to eat you must ask for food … I’m always thinking. You know, when you are not around people and find yourself alone it’s like all those things rewind themselves.’ (Participant 12, F, unemployed)

#### Interpersonal conflict

Interpersonal conflict was reported as a cause and exacerbator for depression. Interpersonal conflict was experienced in different forms, including conflict with the partner (infidelity, domestic violence, parenting issues and financial issues), siblings, extended family, in-laws and parent-child conflict. Participants who reported interpersonal conflict as a causal factor for depression reported conflict with their partner. Participant 5 talked about their experience of interpersonal conflict intersecting with financial difficulties and scarce resources:

‘[… *My husband*] has to provide for his family [of origin] as well since he is the eldest. He would tell me about his sister’s child needs, not considering he also has to provide on this side … when I try to show him what he is doing he responds by saying there’s nothing he can do.’ (Participant 05, F, unemployed)

#### Death, traumatic life experiences and ill health

Death of family member(s) and/or partner was reported as a cause of depression. The participants expressed feelings of abandonment, having no support as a result of the death of a loved one and financial difficulties following the death of a breadwinner or an individual who made financial contributions.

Participants experienced multiple deaths of loved ones, traumatic events and increased socioeconomic responsibilities, and some participants reported loss of the breadwinner. Family members’ ill health and/or participants’ own ill health including being infected with HIV by their partners was reported as causing and exacerbating depression.

Painful life experiences like childhood abuse and rape of a participant’s child were also reported. Participant 13 attributed her depression to being infected with HIV by her husband:

‘What caused my depression is the man at home, he used to have affairs with a lot of women until we got infected with this sickness.’ (Participant 13, F, housewife)

#### Social isolation and self-stigma

Withdrawal from other people and social activities was reported by some participants. Being isolated led to participants feeling worse because of worry. Participant 13 talked about how she chose to isolate herself from social activities and other people because of her anger and how other people perceived her:

‘… I’ve tried to minimize a lot of things that made me seem like a bad person in our stokvel … I told them I will withdraw [*from the stokvel*] and they must continue [*without me*] because at least I can control some of the things that affect me.’ (Participant 13, F, housewife)

### Low self-esteem

A large number of the participants reported low self-esteem. Being depressed impoverished circumstances, and being abandoned by a partner caused low sense of self-worth. Participant 1 expressed self-doubt and concern because she saw herself as unable to leave a financial legacy as well as that of a united family for her children upon her death:

‘Yes, sometimes [*I experience low self-esteem*] l would tell myself if I die my children are going to suffer on their own especially the young ones. I want to leave them in a good state and getting along with other people.’ (Participant 01, F, unemployed)

### Theme 3: Help seeking

The majority of participants reported not seeking help for depressive symptoms because they did not know where to go, the type of available help or that they could get help. A few participants knew about available help but did not seek it because of stigma, with one participant reporting she thought she would get better without help. Participant 16 discussed not knowing how to seek help for depression:

‘… I don’t know where one can go [for help] or how to go about it.’ (Participant 16, F, unemployed)

### Theme 4: Healing and recovery

Participants discussed healing in light of causal factors and symptoms. One participant did not think recovering from depression was possible as she could not identify a tangible cause. A few participants said recovery was possible with help. Although some participants hoped they could recover, they were uncertain about recovery in the absence of change in the perceived causal factors:

‘I think with me it will always be there because I’m staying with someone that can’t change, if he could change then I think I would be the person that I was before.’ (Participant 10, F, unemployed)

### Theme 5: Possible interventions and attitudes to task sharing

Individual, group and couple counselling and income-generating interventions were suggested. The majority of participants were open to task sharing with general health workers trained to provide counselling, while a large number reported they would be willing to join a support group for depression. Participant 13 discussed why she would join a group:

‘I would be meeting with other people and get to talk about our problems. It will help decrease a lot of burden because I’ll be sharing my problems with people.’ (Participant 13, F, housewife)

Nurses, as established and trusted service providers, were suggested as possible service providers who could provide psychoeducation, information on available services and overall direction for getting treatment for depression:

‘Nurses are the ones who enlighten and update us. There is a lot that they help us with … They can tell us in which room the help will be offered and those that want help … can go there.’ (Participant 16, F, umemployed)

Nurse practitioners were identified as being the point of assessment and referral, with existing clinic-based counsellors identified as the cadre to be capacitated to provide a task-sharing manualised counselling intervention under the supervision of district mental health specialists.^35^

## Discussion

As illustrated in Figure 1, the participants identified the main causes of depression as poverty and financial difficulties, painful life experiences and ill health, as well as interpersonal conflict.

Poverty, which is associated with social exclusion, social vulnerability and denial of opportunities and choices,^36^ impacts on people’s sense of agency, rendering them feeling helpless and promoting vulnerability to poor mental health (social causation), which in turn can exacerbate poverty (social drift), thus causing a vicious cycle of poverty and poor mental health.^37,38^

Data from this study support this, revealing that participants expressed a reduced sense of agency, feeling trapped in a cycle of poverty and not being able to transcend this state. This in turn was associated with poor self-esteem and participants feeling unable to deal with daily psychosocial stressors associated with poverty, which included violence, unemployment and insecurity – all of which have been shown to be associated with the development of adult mental illnesses.^36^

This was compounded by the loss of loved ones, which not only caused grief but also led to lowered social support, reduced financial support and increased socioeconomic burdens on dependants left behind. The data also showed that participants in this study experienced multiple interpersonal psychosocial stressors that triggered and compounded their suffering. Research shows that while healthy interpersonal relationships have a beneficial role in the maintenance of psychosocial well-being, poor interpersonal relationships are associated with poorer health outcomes.^39,40^

Interpersonal conflict in the form of intimate partner violence is a particular risk factor for women’s mental health, increasing women’s vulnerability to depression by a factor of two.^41,42^ Violence against women has been highly correlated with depression. In this respect, associated humiliation, an enforced sense of subordination and a sense of having no way out are features often associated with depression.^42,43^

Participants also reported how factors such as worry and social isolation exacerbated their depression (see Figure 1). Worry was expressed as ‘overthinking’ and ‘thinking too much’. Worry was talked about in symbolic terms, pointing to internal dialogue related to experienced problems and habitual brooding or rumination. Evidence shows rumination, a repetitive, passive process of constant mental focus on one’s problems, worsens the experience of depression, as well as serving to maintain it.^44^ The constant self-focused attention and reflection on the experienced distress aggravate negative thinking, impair the ability to problem solve and increase feelings of helplessness and inaction.^44,45^ Participants in the study reported that social isolation made their experience of depression worse, as it made them vulnerable to worrying. Social withdrawal was also a result of self-stigma. Low levels of self-efficacy and reduced self-esteem often result from self-stigma, which is also associated with higher levels of depression.^46^

Through the use of metaphors and explanations that were contextually informed, the participants explained how they understood the symptoms. Kleinman’s explanatory model of illness locates illness and well-being within socio-cultural contexts and refers to how individuals make meaning of their illness.^10^ The experience of depression and its understanding are mediated by cultural meanings and experiences. This affects if and how depression is reported, treatment sought, service user-service provider interaction and how treatment is received.^10^ Ganasen et al. argue that mental health literacy can be shaped by knowledge and beliefs derived from other sources such as cultural and personal beliefs.^11^

The findings in this study both confirm and expand upon research in similar resource-scarce settings. Several studies reported that causal factors of depressive symptoms were focused around socioeconomic and interpersonal problems even though they could not recognise the constellation of their symptoms as depression.^27,47,48,49,50^

In their thematic synthesis of qualitative research in sub-Saharan Africa by Mayston et al., causal influences of depression were associated with social adversity, primarily economic issues and interpersonal conflict.^27^ This suggests the need for depression treatment goals in these contexts to focus on reconstructing the person’s life and help them develop healthy coping skills.

In relation to pathways to care, participants reported not seeking help for the symptoms of depression because of poor mental health literacy. Similarly, Andersson et al.’s study in the Eastern Cape in South Africa revealed that in addition to financial constraints, poor mental health literacy was a barrier to help seeking for depression.^9^ They found that barriers to help seeking were that: people thought they should be able to deal with problems on their own, expected the problems to disappear on their own or were too embarrassed to discuss their problems with anyone and fear of being discriminated against for seeking help for ‘mental health’ problems.^9^

Further, in a study looking at structural and attitudinal barriers to mental health care and predictors of treatment dropout in South Africa, the most commonly cited reason for not seeking care was a low perceived need for treatment.^51^ In addition, Hlongwa and Sibiya^52^ address the need to understand challenges in implementing policies, such as integrating mental health care into PHC, in order to address barriers to help seeking for mental health services. Participants highlighted the need for information on the availability of services for depression, underscoring the importance of psychoeducation interventions to improve mental health literacy among chronic care service users. Psychoeducation interventions in the form of lectures, simulations, group discussions and role plays have been shown to improve help seeking and openness to counselling interventions in Kenya.^53^

The success of task-shared interventions that help people to cope with these identified causal factors of depression (i.e. poverty and financial difficulties, worry, interpersonal conflict, death, painful life experiences and ill health and social isolation and self-stigma) using cognitive behavioural therapy (CBT) interventions such as behavioural activation, healthy thinking and problem-solving therapy in sub-Saharan Africa is supported by a number of research studies.^54,55,56,57,58,59^

### Implications for practice

It is unlikely that Setswana-speaking adults with chronic conditions and comorbid depression will simply present to their primary care providers to discuss their depression according to existing clinical criteria. Non-Setswana clinicians may need to contextualise their approach to the explanatory model discussed in this study. For example, asking a metaphorical question about the heart may yield information on emotional well-being or lack thereof. Furthermore, interventions acceptable to the participants were identified as those that could help them cope with causal factors. Psychoeducation also emerged as important to improve mental health literacy and should assist with identification and help-seeking behaviour,^12^ but cultural congruency is important to promote engagement. In this regard, the findings of this study help promote greater understanding of how depression is understood within the Setswana-speaking population of South Africa.

### Limitations of the study

The study was limited to participants from a particular geographic and cultural and linguistic group in South Africa. Generalisability of the findings to other cultural groups is thus limited, and the intervention informed by this formative research will require adaptation to ensure cultural congruence with other cultural/linguistic groups. Although the gender bias in this study is reflective of local PHC settings,^32^ it might be challenging to generalise the results to a larger population. It was not possible to determine whether the participants’ level of education had an impact on their mental health literacy. The study may have benefited from member checking by providing participants with the opportunity to engage with and add to the interpreted data.

## Conclusion

This study provides a comprehensive understanding of the various factors that contribute to the development and exacerbation of depression in this Setswana-speaking sub-population of South Africa. It was developed with rich contextual information that focused on perceived causes, exacerbators, symptoms of depression, course of the condition and help seeking. The emerging explanatory model of depression for the Setswana-speaking sub-culture was similar to that of other contexts in South Africa in terms of the well-known social determinants of depression.^27,47,48,49,50^ Poverty and financial difficulties were the most reported causes of depression. The study highlights the need for psychoeducation to help service users report their symptoms and know where to get help.
